# A pretreatment TNM-metabolic vulnerability score with EBV DNA adjustment for risk stratification in non-metastatic nasopharyngeal carcinoma

**DOI:** 10.3389/fonc.2026.1914286

**Published:** 2026-10-30

**Authors:** Jinghua Zhong, Chunyan Zhou, Qiang Yi, Kui Zhong, Zheng Chen, Xinting Ouyang, Changyong Xu, Jiahua Liao, Weifeng Chen, Xiaojun Zhang

**Affiliations:** 1 Department of Oncology, The First Affiliated Hospital of Gannan Medical University, Ganzhou, China; 2 The First Clinical Medical College, Gannan Medical University, Ganzhou, China; 3 Department of Oncology, Ma’anshan 17th Metallurgical Hospital, Ma’anshan, China; 4 Department of Otorhinolaryngology Head and Neck Surgery, First Affiliated Hospital of Gannan Medical University, Ganzhou, China

**Keywords:** BMI, bootstrap validation, EBV DNA, metabolic vulnerability, nasopharyngeal carcinoma, ridge Cox, THR

## Abstract

**Background:**

TNM stage and pretreatment plasma Epstein-Barr virus (EBV) DNA are central to risk assessment in nasopharyngeal carcinoma (NPC), but they do not fully capture host metabolic reserve.

**Methods:**

We retrospectively analyzed 160 patients with non-metastatic NPC treated from 2013 to 2022. The integrated metabolic vulnerability score (IMVS; range, 0-3) assigned 1 point each for stage IVA, triglyceride-to-HDL cholesterol ratio <1.217, and body mass index <23.79 kg/m2. Cox regression and bootstrap internal validation evaluated progression-free survival (PFS), with EBV DNA included as an adjustment variable. A broader 271-patient cohort from the same institution was used only for internal sensitivity analysis.

**Results:**

Thirty-three PFS events occurred in the modeling cohort. Five-year PFS was 100.0%, 91.9%, 72.1%, and 50.0% for IMVS scores 0, 1, 2, and 3, respectively. IMVS remained associated with PFS after adjustment for EBV DNA status (HR, 2.74; bootstrap percentile 95% CI, 1.89-4.75; P<0.001), although EBV DNA status itself was not independently significant in this cohort. The optimism-corrected AUC was 0.715 for stage IVA, THR, and BMI and 0.727 after adding EBV DNA. In the broader same-institution cohort, the association persisted but was attenuated (HR, 1.55; 95% CI, 1.14-2.10; P = 0.005).

**Conclusion:**

IMVS is an exploratory, low-cost risk-stratification tool that may complement, rather than replace, TNM stage and plasma EBV DNA. Prospective external validation and recalibration of the data-derived thresholds are required before clinical use.

## Introduction

Nasopharyngeal carcinoma (NPC) shows a marked geographic pattern and is still a common clinical problem in endemic areas, particularly in Southern China. Modern radiotherapy and combined systemic treatment have improved outcomes, yet recurrence and distant metastasis remain familiar problems in daily practice, even among patients with apparently similar stage and treatment (1–13).

In routine care, TNM stage and pretreatment plasma Epstein-Barr virus (EBV) DNA are two important anchors for estimating risk. Plasma EBV DNA has demonstrated value in tumor burden assessment, residual-disease monitoring, and prognostication (14–25). Its performance is nevertheless context-dependent. In a non-endemic cohort of 167 patients with EBV-related NPC, a predictive pretreatment threshold could not be established and isolated positive follow-up results were not prognostic; an earlier Western study of 36 patients reported 71.3% sensitivity for recurrence detection (26, 27). These studies support the use of EBV DNA, while illustrating why detection time point, threshold, assay, and population matter. Additional markers may help characterize risk that is not captured by anatomic extent or viral measurements alone.

One possible source of this unexplained heterogeneity is host metabolic reserve. TNM stage describes anatomic extent, whereas body mass index (BMI) and circulating lipids may reflect nutritional state, inflammation, and the systemic consequences of disease. A low BMI could indicate reduced reserve or cancer-associated weight loss; a low triglyceride-to-HDL cholesterol ratio (THR) could reflect altered lipid handling or inflammatory remodeling. Tumor cells also require lipid and other metabolic substrates for growth and stress adaptation (28–45). These are plausible but distinct explanations: a blood lipid ratio is not a direct measure of tumor metabolism, and this retrospective study cannot establish whether the measured profile causes NPC progression or arises as a consequence of it.

The original thesis suggested that THR and BMI were associated with NPC prognosis, and that a multivariable nomogram had moderate discrimination. For this manuscript, we deliberately chose a simpler route: a small point score that could be calculated at the bedside, followed by several sensitivity analyses to show where the signal was stable and where it was not.

Recent NPC studies make this approach clinically plausible. Wu et al. showed that EBV DNA combined with LA, an immune-nutritional marker defined as lymphocytes x albumin, improved prognostic stratification; Xiao et al. found that pretreatment TG/HDL-C, TyG, and TyG-BMI predicted PFS in locally advanced NPC; and Jiayu Zhou et al. reported a prognostic serum metabolomic signature (29–31). Together with prior NPC lifestyle and BMI studies, these findings suggest that tumor burden and host reserve may need to be read together (43–45). We therefore developed IMVS to combine anatomic tumor burden with routinely available indicators of host metabolic reserve. Its intended role is preliminary risk exploration using measurements commonly available before treatment; it complements rather than replaces TNM stage or plasma EBV DNA.

## Materials and methods

### Patients and data source

We retrospectively screened 1200 consecutive patients with stage I-IVA NPC treated at the First Affiliated Hospital of Gannan Medical University from January 1, 2013 to January 1, 2022. Of these, 529 were excluded because of concomitant malignancy or lipid-altering comorbidity, 299 because of incomplete clinical data, 203 because standardized radical treatment was not completed, 3 because stage I disease was treated surgically, and 6 because of loss to follow-up, leaving 160 patients in the modeling cohort (**Figure 1**). Tumor stage was harmonized according to the eighth edition of the AJCC/UICC staging system. Patients diagnosed before its implementation were retrospectively restaged from the original clinical examination, endoscopic, and imaging records. All included patients had non-metastatic, non-keratinizing NPC. Follow-up ended on December 31, 2022.

**Figure 1:**
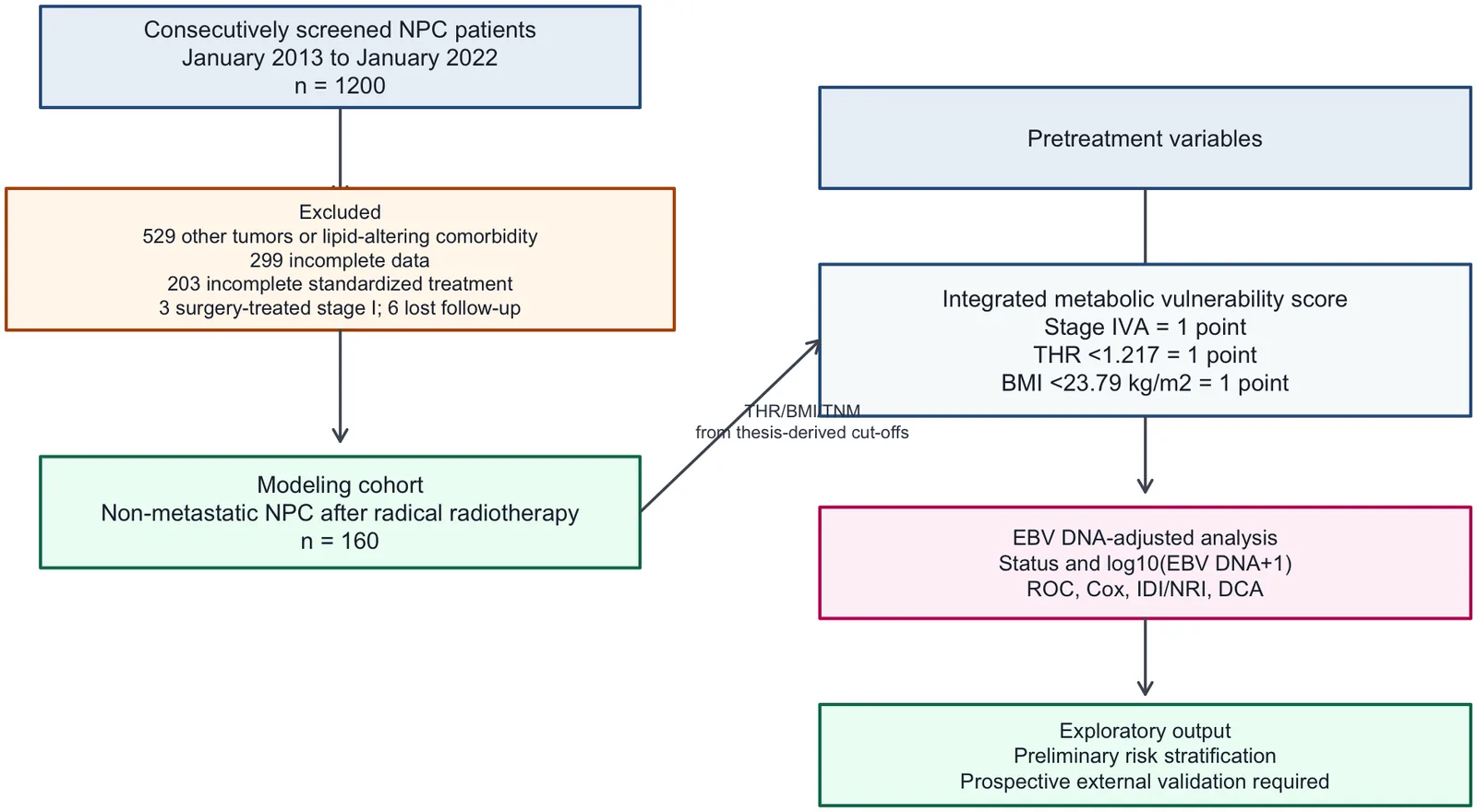
Patient selection and IMVS construction.

The expanded internal sensitivity cohort was assembled from the same institutional raw-data environment and included 271 patients with available stage, BMI, THR, EBV DNA, and PFS fields. Because it overlapped with the modeling cohort in calendar period and data source, we did not regard it as an external or temporal validation cohort. We used it only to examine whether the main direction of association remained visible under broader, less selected data conditions.

### Score construction and EBV DNA processing

THR was calculated as triglycerides divided by HDL-C, a lipid index related to insulin resistance and atherogenic metabolic status in non-cancer populations and increasingly evaluated in cancer prognosis (30, 40–42). No accepted NPC-specific THR threshold is available. We used THR <1.217 and BMI <23.79 kg/m2 from ROC/Youden analyses performed in the same modeling cohort. These thresholds are exploratory and may yield optimistic performance estimates; they should not be interpreted as established clinical cut-offs and will require independent validation and, if necessary, recalibration. Stage IVA was used as the anatomic component because stage II cases were few and binary stage IVA performed similarly to ordinal stage while keeping the score easy to use. IMVS assigned 1 point each for stage IVA, low THR, and low BMI.

Pretreatment EBV DNA was extracted from the original clinical EBV field. Negative or below-detection entries were coded as 0 copies/mL, and mantissa-exponent entries were converted to copies/mL. PCR assay choice and detection thresholds can affect EBV DNA measurement, and prior NPC studies have used EBV DNA both as a continuous and a categorical biomarker (14–20, 24). In our records, PCR platform, assay kit, and lower limit of detection were not consistently documented. For that reason, EBV DNA was analyzed conservatively as positive/negative and as log10(EBV DNA + 1).

### Statistical analysis and sensitivity checks

PFS was measured from completion of radiotherapy to recurrence, metastasis, death, or last follow-up; death was therefore included in the composite endpoint rather than treated as a competing event for PFS. OS was measured to death or last follow-up. Because only 33 PFS events were available, Cox models were kept deliberately small, in line with events-per-variable concerns in prognostic modeling (48, 49). We then examined the stability of the findings using 1000-resample bootstrap percentile intervals for key HRs, optimism-corrected AUC estimates, ridge Cox sensitivity analysis, VIF diagnostics, continuous THR/BMI sensitivity analyses, calibration assessment, and an exploratory 0–4 EBV-IMVS score. Discrimination, incremental value, decision-curve analysis, calibration, internal validation, and reporting were interpreted with reference to established prediction-model methods and TRIPOD guidance (46–53). Recurrence-specific Fine-Gray modeling was not performed because cause-specific progression and non-cancer death fields were not available.

## Results

### Cohort profile and follow-up

The modeling cohort included 160 patients. Median observed PFS follow-up was 73.0 months (IQR, 44.8-82.2), and reverse KM median follow-up was 77.0 months. EBV DNA was positive in 57 patients (35.6%) (**Table 1**).

**Table 1:** Baseline characteristics of the modeling cohort.

Characteristic	Value
Patients, n	160
Male sex, n (%)	113 (70.6)
Age, years, mean (SD)	51.8 (10.3)
Stage II/III/IVA, n	8/58/94
EBV positive, n (%)	57 (35.6)
Concurrent chemoradiotherapy, n (%)	150 (93.8)
BMI, kg/m2, mean (SD)	22.75 (2.87)
THR, mean (SD)	1.03 (0.35)
PFS events, n (%)	33 (20.6)
Deaths, n (%)	33 (20.6)

### Primary IMVS performance

IMVS separated patients into ordered PFS strata. The two extreme groups were small, especially score 3, so these estimates should be read as signals rather than precise risk estimates (**Table 2**; **Figure 2**).

**Table 2:** IMVS construction and 5-year PFS strata.

Score/component	N or definition	5-year PFS	95% CI	PFS events, n
IMVS components	Stage IVA + THR <1.217 + BMI <23.79 kg/m2	1 point each	Range 0–3	–
0	20	100.0%	100.0%-100.0%	0
1	62	91.9%	85.4%-99.0%	7
2	66	72.1%	61.9%-84.0%	20
3	12	50.0%	28.4%-88.0%	6

**Figure 2:**
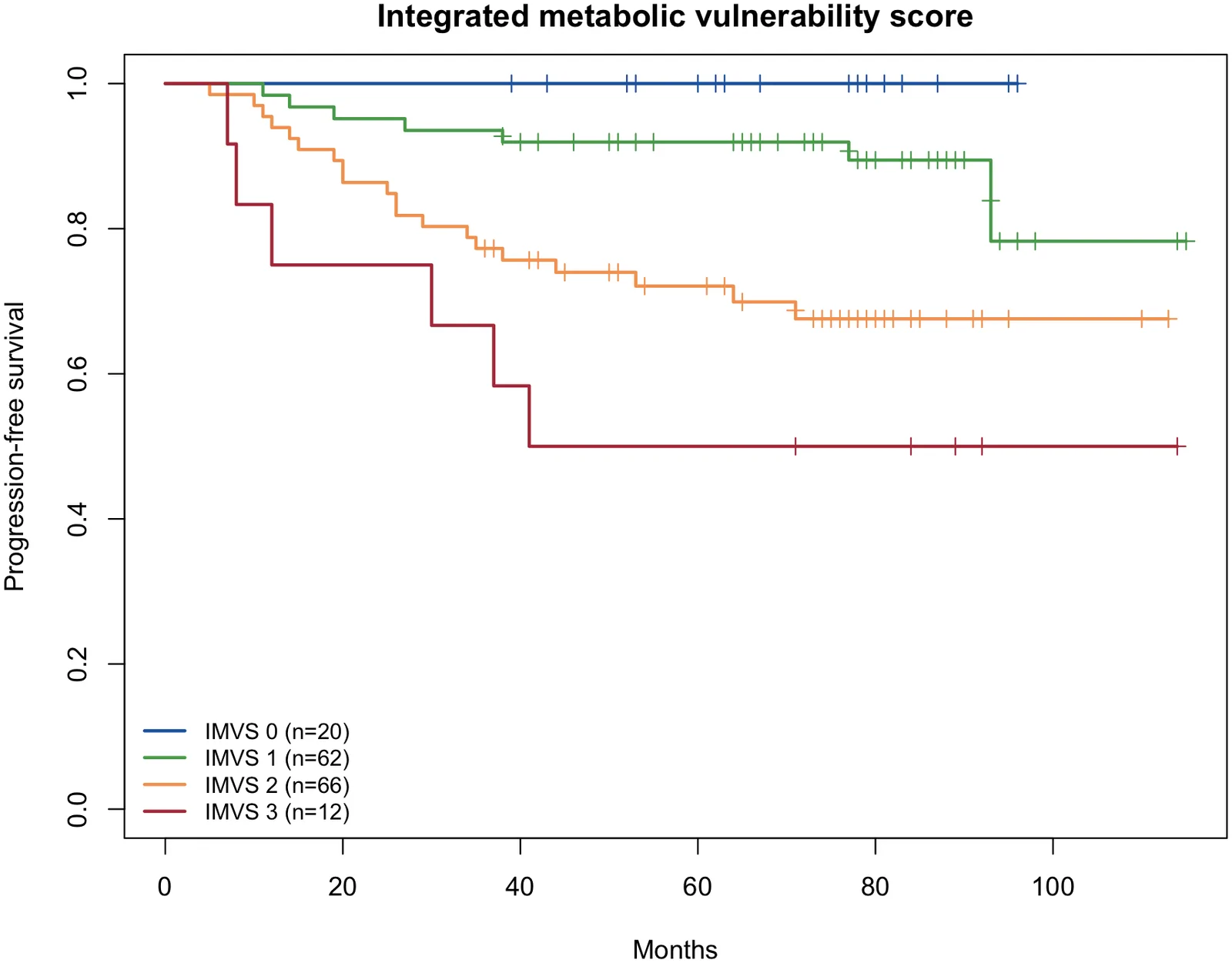
Kaplan-Meier curves by IMVS. Score 0 had no observed PFS events and should be interpreted as an unstable small-sample stratum.

### Overfitting, stage encoding, and EBV-inclusive score sensitivity

The IMVS association was broadly preserved in bootstrap and ridge analyses. The low-THR component, however, had an unstable upper bootstrap interval because sparse events led to separation in some resamples. VIF values did not suggest meaningful collinearity among stage IVA, low THR, low BMI, and EBV DNA status (**Tables 3**, **Table 4**).

**Table 3:** Robustness analyses requested during major revision.

Analysis	Estimate	Bootstrap/sensitivity result	P value
IMVS adjusted for EBV DNA status	2.74	1.89-4.75	<0.001
EBV-IMVS 0–4 score	2.23	1.56-3.59	<0.001
Low THR adjusted for stage/BMI/EBV DNA	5.49	1.99-307822876.69	0.021
Ridge Cox: ridge(imvs)	2.64	penalized sensitivity	
Ridge Cox: ridge(ebv_dna_positive)	1.41	penalized sensitivity	
VIF: stage_iva	1.04	no severe collinearity	
VIF: low_thr	1.05	no severe collinearity	
VIF: low_bmi	1.05	no severe collinearity	
VIF: ebv_dna_positive	1.04	no severe collinearity	

**Table 4:** Apparent AUC, optimism-corrected AUC, and nonparametric bootstrap 95% CI.

Model	Apparent AUC	Optimism-corrected AUC	AUC 95% CI
TNM stage ordinal	0.651	0.65	0.572-0.722
Stage IVA binary	0.645	0.644	0.563-0.719
Stage IVA + THR + BMI	0.731	0.715	0.647-0.808
IMVS	0.725	0.724	0.645-0.800
IMVS + EBV DNA	0.738	0.727	0.646-0.821
EBV-IMVS 0–4 score	0.719	0.718	0.624-0.804

### EBV DNA handling and 0–4 EBV-IMVS

EBV DNA status alone was not independently significant in this cohort, a finding that differs from much of the NPC literature. The most likely explanations are limited sample size, retrospective assay heterogeneity, and incomplete documentation of detection standards. Adding EBV DNA directly to create a 0–4 EBV-IMVS produced a graded pattern, but it did not improve discrimination over the simpler IMVS with EBV adjustment; the 4-point group contained only 5 patients (**Table 5**; **Figure 3**).

**Table 5:** Exploratory EBV-IMVS 0–4 score.

EBV-IMVS	N	5-year PFS	95% CI
0	13	100.0%	100.0%-100.0%
1	52	90.4%	82.7%-98.8%
2	55	84.8%	75.5%-95.1%
3	35	62.9%	48.7%-81.1%
4	5	40.0%	13.7%-100.0%

**Figure 3:**
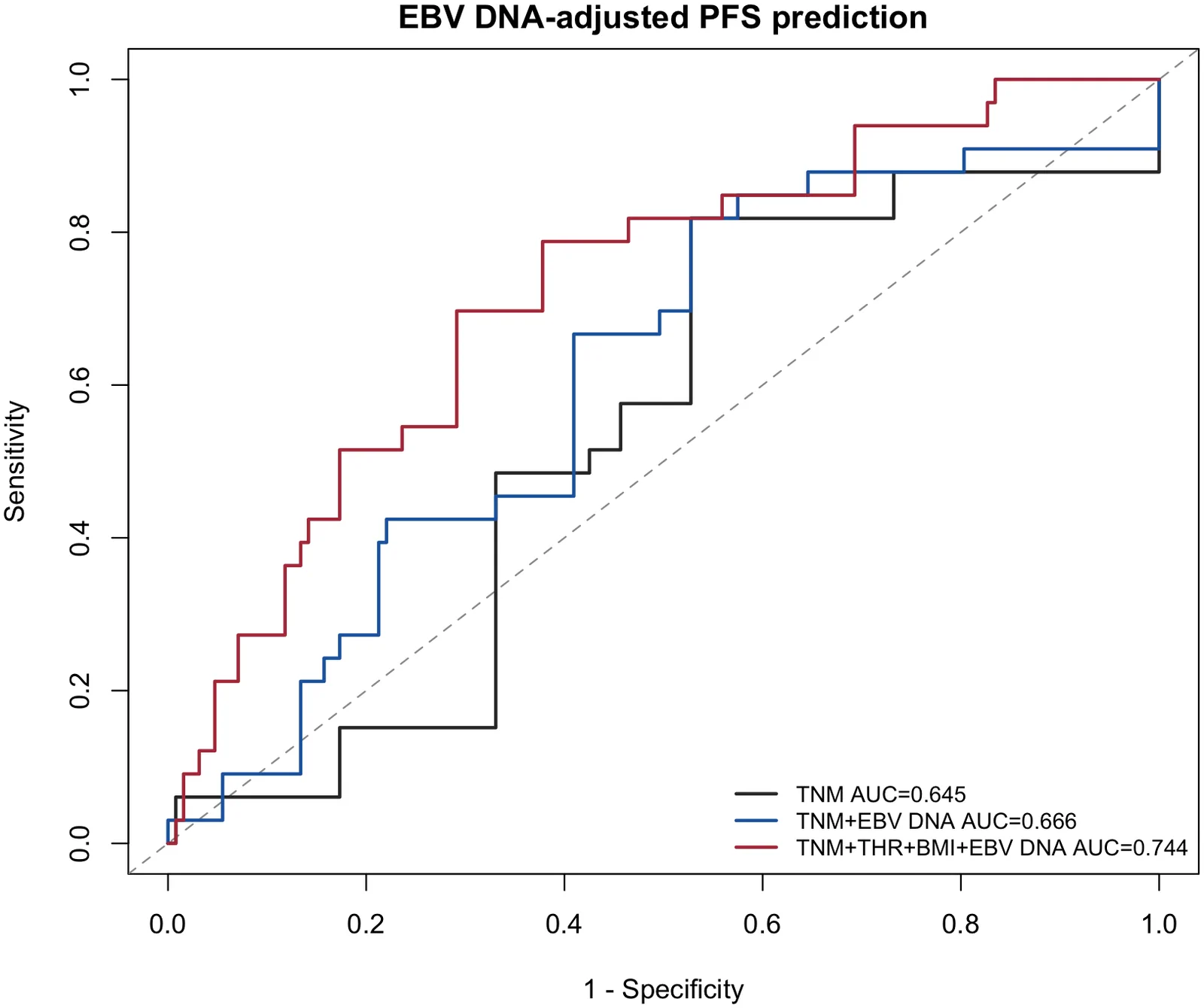
ROC curves after EBV DNA integration.

### Expanded internal cohort and stage migration

The expanded internal cohort was broadly similar to the modeling cohort for age, sex, stage IVA rate, EBV DNA positivity, BMI, and PFS event rate, but THR was higher and more variable. Since this cohort came from the same institution and overlapping years, its attenuated gradient should not be read as external validation. It more likely reflects broader eligibility, measurement heterogeneity, or lipid-assay/batch variation. IMVS remained significant after EBV DNA adjustment, but the absolute 5-year PFS separation was smaller (**Tables 6**, **7**; **Figures 4**, **5**).

**Table 6:** Modeling cohort versus expanded internal sensitivity cohort.

Characteristic	Modeling cohort	Expanded cohort
N	160	271
Age, years, mean (SD)	51.8 (10.3)	50.8 (10.5)
Male sex, n (%)	113 (70.6%)	194 (71.6%)
Stage IVA, n (%)	94 (58.8%)	148 (54.6%)
EBV DNA positive, n (%)	57 (35.6%)	100 (37.5%)
BMI, kg/m2, mean (SD)	22.8 (2.9)	22.9 (3.1)
THR, mean (SD)	1.03 (0.35)	1.40 (1.17)
PFS events, n (%)	33 (20.6%)	55 (20.3%)
Median PFS follow-up, months	73.0 (44.8-82.2)	77.0 (54.5-86.0)

**Table 7:** Stage encoding and score comparison.

Analysis	HR (95% CI)	P value	AIC
Stage ordinal per level	3.32 (1.43-7.70)	0.005	311.3
Stage IVA vs II-III	3.48 (1.44-8.44)	0.006	312.5
IMVS per point	2.74 (1.73-4.34)	<0.001	302.6
EBV-IMVS per point	2.23 (1.52-3.25)	<0.001	304.0

**Figure 4:**
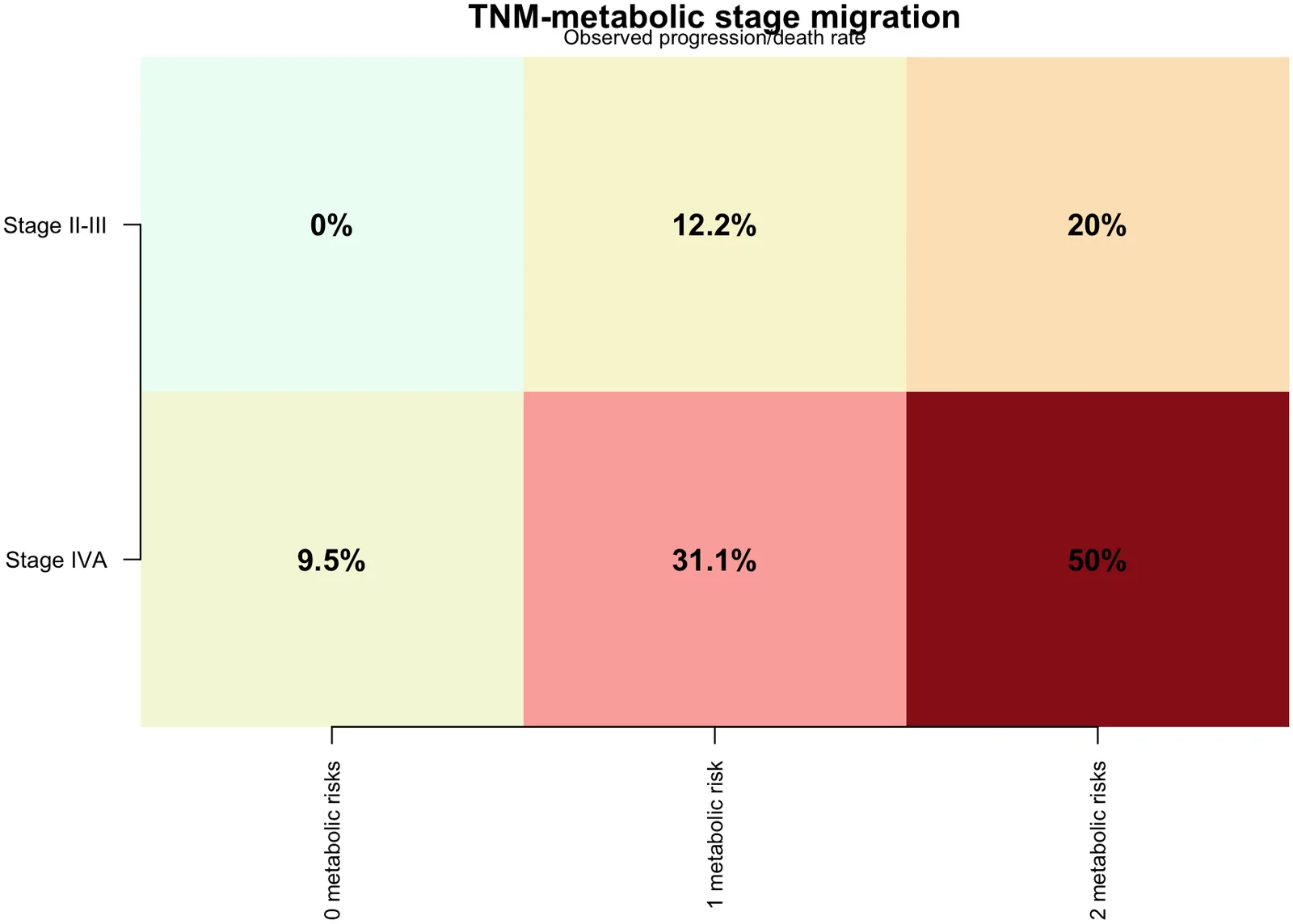
Revised TNM-metabolic stage migration heatmap.

**Figure 5:**
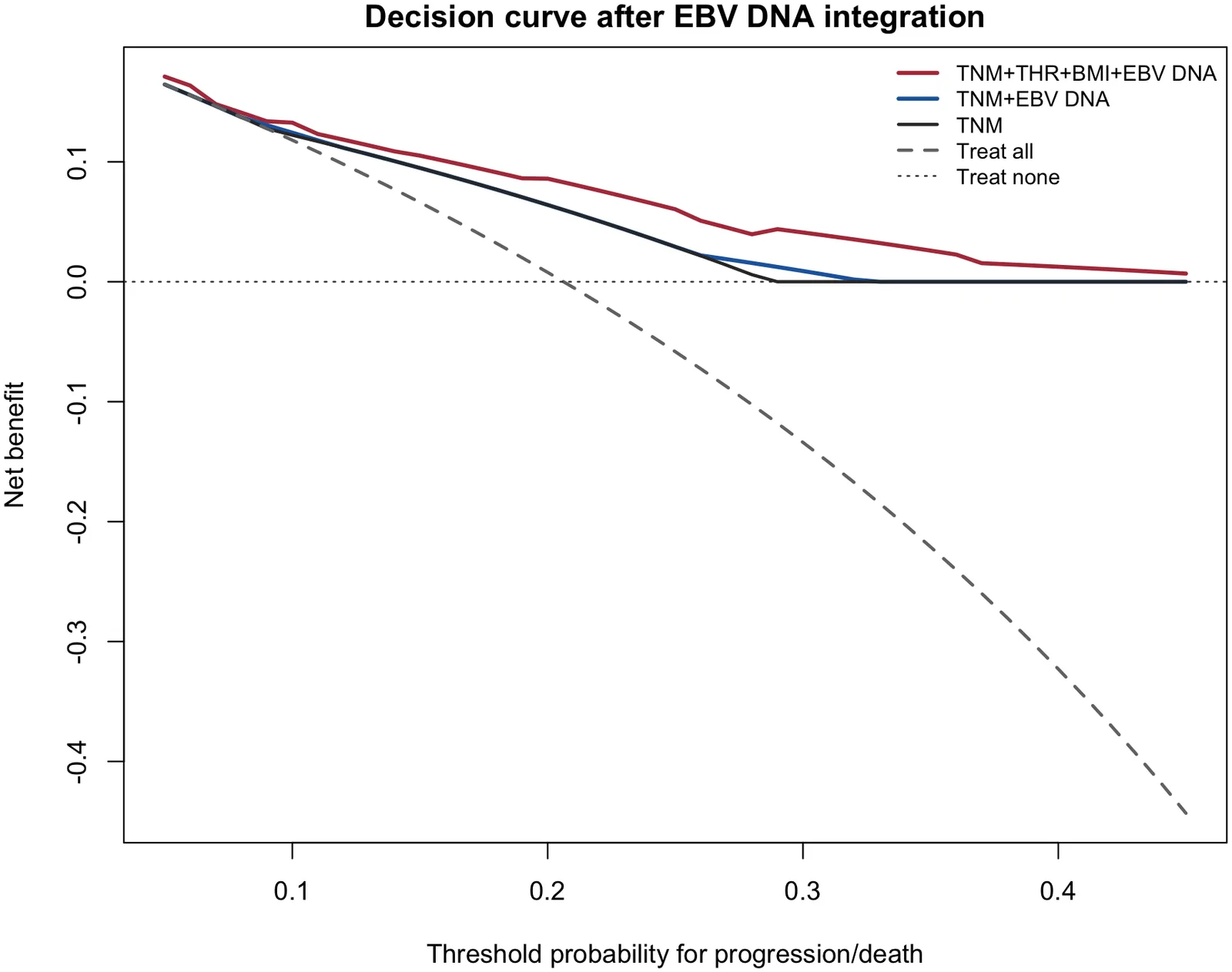
Decision curve analysis after EBV DNA integration.

### Cut-off sensitivity and model calibration

Because the THR and BMI thresholds were data-driven, we also modeled both variables continuously. The directions were consistent with the main analysis but were not statistically significant. This pattern is compatible with a threshold-like vulnerability phenotype rather than a simple linear dose-response. In practical terms, small BMI differences within the usual range may matter less than crossing into a low-reserve state. The binary clinical-metabolic-EBV model had a numerically higher AUC than the continuous THR/BMI model, although the bootstrap AUC difference was not significant (**Table 8**; **Figure 6**).

**Table 8:** Cut-off sensitivity, AUC comparison, and calibration.

Analysis	Estimate	P value/interval	Interpretation
Continuous THR per SD adjusted for stage/BMI/EBV	0.73 (0.50-1.07)	0.106	Direction supports lower THR as adverse, but not significant as a linear term
Continuous BMI per SD adjusted for stage/THR/EBV	0.94 (0.64-1.37)	0.738	Direction supports lower BMI as adverse, but not significant as a linear term
Continuous model AUC	0.700	apparent AUC	Compared with binary clinical-metabolic-EBV model AUC 0.744
IMVS+EBV DNA vs EBV-IMVS 0-4	0.02	0.002-0.037; P = 0.039	Bootstrap paired AUC difference
Binary stage/THR/BMI/EBV vs continuous THR/BMI model	0.045	-0.011-0.098; P = 0.108	Bootstrap paired AUC difference
Hosmer-Lemeshow grouped calibration	1.33	df=2; P = 0.513	4 groups

**Figure 6:**
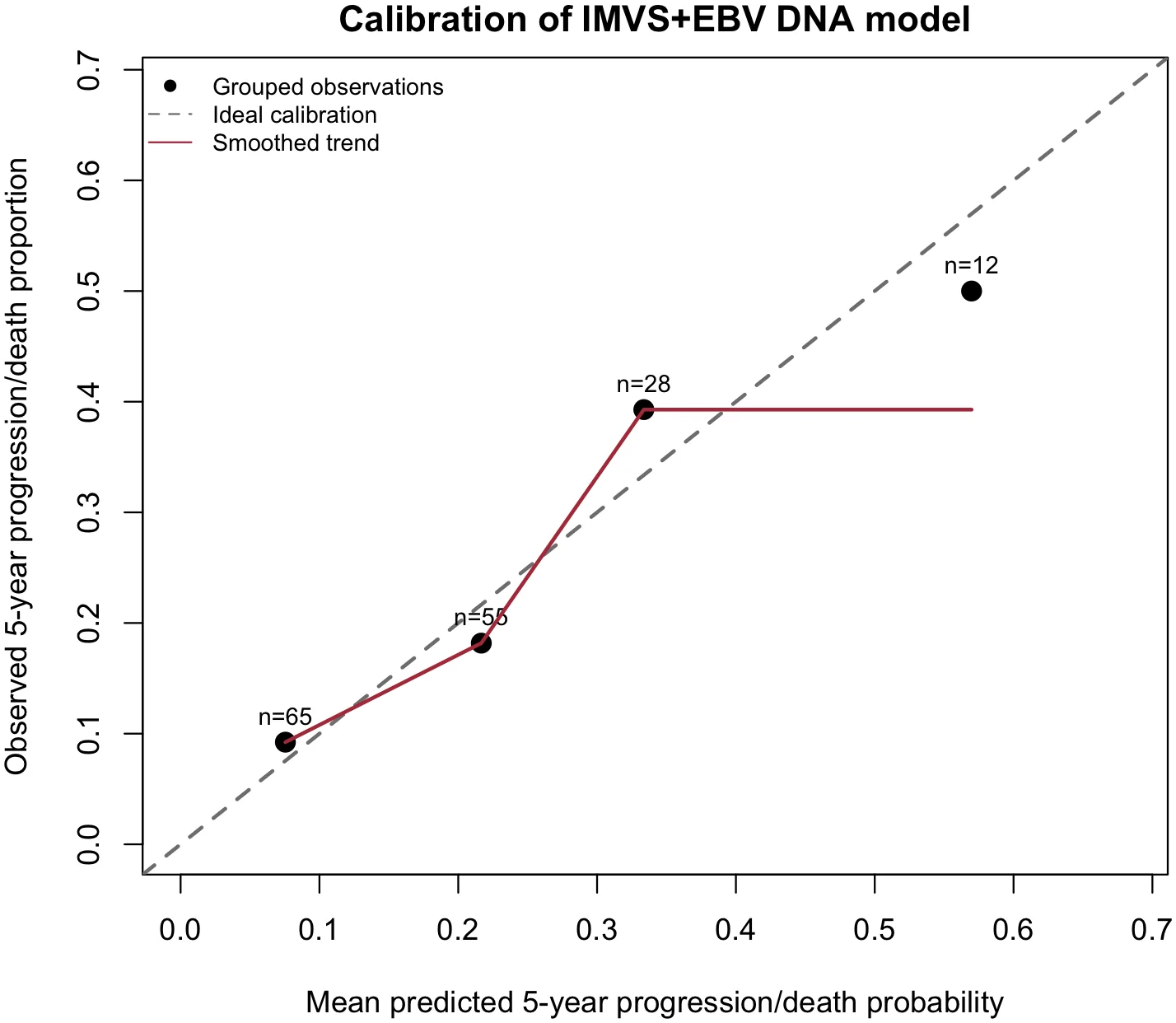
Grouped calibration plot for the IMVS+EBV DNA model.

## Discussion

In this single-center cohort, IMVS combined tumor burden, represented by stage IVA, with two routinely available indicators of host metabolic reserve. Its potential practical appeal is that BMI and a routine lipid profile are available before treatment. IMVS is investigational and complements rather than replaces TNM stage or plasma EBV DNA. IMVS separated patients into ordered PFS strata and remained associated with outcome after adjustment for EBV DNA. The result is an early signal, not proof of a finished prognostic model. Only 33 PFS events were observed, score 0 had no events, and score 3 included 12 patients; together with the data-derived thresholds, these features limit precision and raise the possibility of optimistic estimates (48–53).

Biologically, a low BMI and low THR might accompany reduced nutritional reserve, inflammatory lipid remodeling, or disease-associated catabolism. These processes could plausibly coexist with a more aggressive clinical course or reduced tolerance of intensive treatment, while TNM stage describes anatomic spread rather than systemic condition (28–45). The observed association does not demonstrate such a pathway: no tissue metabolic assays, inflammatory markers, or longitudinal weight and lipid measurements were available. Nor does it prove additional prognostic information beyond TNM, since stage IVA is itself part of the score. Mechanistic and stage-stratified studies are needed to separate host vulnerability from tumor-driven metabolic change.

The study fits with a growing NPC literature in which host-derived biomarkers are used alongside TNM stage and EBV DNA (14–31). Wu et al. reported that low LA combined with EBV DNA positivity identified patients with poor outcomes, suggesting that tumor burden and host reserve can carry different information (29). IMVS follows the same broad clinical logic but uses metabolic rather than immune-nutritional variables. The optimism-corrected AUC was 0.727. Direct comparison with discrimination estimates from other cohorts and models is inappropriate because populations, endpoints, and validation methods differ.

Metabolomic work also supports the idea that systemic metabolic status contains prognostic information. Jiayu Zhou et al. reported a 9-metabolite serum signature with C-indexes of 0.71 and 0.73 in training and validation cohorts, and a marked difference in 5-year PFS between high- and low-risk groups (31). Such studies are mechanistically rich, but metabolomic profiling is not yet available in many routine clinics. IMVS was designed with a different aim: to approximate part of that host metabolic signal using data that are already collected before treatment.

We used stage IVA as the anatomic component because the cohort contained few stage II patients and because binary stage IVA performed similarly to ordinal stage while keeping the score easy to calculate. This choice is pragmatic rather than definitive. Larger external datasets should reassess whether stage III deserves separate weighting or whether stage should be modeled more flexibly.

The exploratory 0–4 EBV-IMVS asked a straightforward question: should EBV DNA be built directly into the point score? In this dataset, the answer was not compelling. The 0–4 score showed an ordered pattern, but it did not improve discrimination over IMVS with EBV adjustment, and the highest category included only 5 patients. We therefore kept IMVS as the primary score and treated EBV DNA as an adjustment variable.

The EBV DNA result should be interpreted carefully. EBV DNA was not independently significant here, which differs from landmark NPC studies (14–20, 24). Non-endemic studies also show that biomarker interpretation varies with assay, sampling time, and population: the LEA study found no predictive pretreatment threshold, while an earlier Western cohort showed useful but incomplete sensitivity for detecting recurrence (26, 27). These observations do not diminish the established value of plasma EBV DNA. In our cohort, incomplete assay metadata, modest sample size, and limited events may have reduced precision; we cannot infer that IMVS performs better than, or can replace, serial standardized EBV DNA assessment.

The low-THR finding is clinically interesting but should not be overread. One possible explanation is tumor-associated metabolic consumption, inflammatory lipoprotein remodeling, and reduced host reserve. Cancer cells remodel glucose, amino-acid, and lipid metabolism to support proliferation, membrane synthesis, stress adaptation, and survival (32–39). At the same time, low THR showed separation instability in bootstrap resampling, so its isolated HR is less reliable than the combined IMVS result.

Our result differs from Xiao et al., who found worse PFS in patients with higher TG/HDL-C in locally advanced NPC (30). The two findings may be describing different parts of the metabolic spectrum. Our threshold selected a very low THR phenotype, possibly reflecting lipid depletion and vulnerability, whereas high-ratio definitions may capture insulin resistance and atherogenic metabolic dysfunction (40–42). Xiao et al. also reported non-linear relationships between TG/HDL-C-related indices and PFS, which is consistent with the weaker continuous-variable effects observed here (30).

The broader 271-patient analysis was drawn from the same hospital and overlapping calendar years and was therefore not an external validation cohort. It served only as a same-institution sensitivity analysis. The direction of association persisted, but the absolute gradient and effect size were attenuated. Its higher and more variable THR distribution points to broader eligibility, cohort heterogeneity, or lipid measurement variation. These findings support further study of IMVS but do not establish transportability to other institutions or populations.

Viewed together with LA, TG/HDL-C/TyG, metabolomic, lifestyle, and BMI studies, IMVS belongs to a broader tumor-host risk-stratification approach (29–31, 43–45). Its main advantage is not complexity, but ease of use. It can be calculated from stage, lipid profile, and BMI before treatment, which may be helpful in clinics where rapid risk enrichment is needed without specialized assays or nomogram software. This integrated approach is consistent with the CACA guidelines, which emphasize multidisciplinary care, clinical staging, and nutritional assessment in nasopharyngeal carcinoma (54).

A reasonable next step is prospective evaluation of whether IMVS can identify a subgroup for closer surveillance and early nutritional or metabolic assessment. Such use should be studied alongside standard TNM staging and serial EBV DNA rather than as a substitute for either measure. The present data do not justify changing surveillance intervals or treatment intensity.

### Limitations

This study has several limitations. It was retrospective and single-center, and only 33 PFS events were available. The score 0 group had no events, whereas the score 3 group contained only 12 patients and 6 events; overfitting and imprecise survival estimates therefore remain possible despite bootstrap, ridge, calibration, and sensitivity analyses (48–53). The score includes stage IVA, so its association does not establish incremental prognostic value beyond TNM staging. BMI and THR were measured before treatment, but we did not assess longitudinal metabolism, tumor metabolic activity, inflammatory mediators, treatment tolerance, or causal mechanisms. Thus, we cannot establish whether the profile drives disease, reflects tumor aggressiveness, or represents a host response. EBV DNA was not independently significant in this cohort, and PCR platform, assay kit, and detection limit were incompletely documented; standardized measurements at defined time points are needed. The 271-patient analysis used overlapping years and the same institutional source and therefore provides internal sensitivity evidence, not external validation. The THR and BMI thresholds were derived in the modeling cohort and may be optimistic. Our findings should not guide treatment or surveillance. External cohorts spanning endemic and non-endemic settings must test discrimination, calibration, incremental value over TNM and EBV DNA, and any required threshold recalibration before clinical use. PFS included death as a composite event; recurrence-specific competing-risk analysis was unavailable because cause-specific progression and non-cancer death fields were incomplete. We also lacked toxicity, treatment-interruption, and post-treatment EBV DNA data.

## Conclusion

IMVS is an exploratory score that combines stage with BMI and THR and may reflect host-related risk not fully summarized by anatomic extent. Its biological meaning, incremental clinical value, and transportability remain unproven; prospective external validation and recalibration are required before it can inform care.

## Data Availability

The datasets generated and analyzed during the current study are not publicly available because they contain institutional retrospective clinical data, but de-identified data may be available from the corresponding authors on reasonable request and subject to institutional and ethical approval.
